# The Future of Artificial Intelligence Using Images and Clinical Assessment for Difficult Airway Management

**DOI:** 10.1213/ANE.0000000000006969

**Published:** 2025-01-10

**Authors:** Silvia De Rosa, Elena Bignami, Valentina Bellini, Denise Battaglini

**Affiliations:** From the * Centre for Medical Sciences – CISMed, University of Trento, Trento, Italy; † Anesthesia and Intensive Care, Santa Chiara Regional Hospital, APSS Trento, Trento, Italy; ‡ Anesthesiology, Critical Care and Pain Medicine Division, Department of Medicine and Surgery, University of Parma, Parma, Italy; § Anesthesia and Intensive Care, IRCCS Ospedale Policlinico San Martino, Genova, Italy.

## Abstract

Artificial intelligence (AI) algorithms, particularly deep learning, are automatic and sophisticated methods that recognize complex patterns in imaging data providing high qualitative assessments. Several machine-learning and deep-learning models using imaging techniques have been recently developed and validated to predict difficult airways. Despite advances in AI modeling. In this review article, we describe the advantages of using AI models. We explore how these methods could impact clinical practice. Finally, we discuss predictive modeling for difficult laryngoscopy using machine-learning and the future approach with intelligent intubation devices.

Difficult airway management is a scenario in which the clinician encounters challenges with oxygenation, ventilation, laryngoscopy, and/or tracheal intubation.^1^ Several algorithms for difficult airway management and prediction exist, although current protocols demonstrate low sensibility and specificity.^2^ One-size-fits-all solutions are ill-suited for complex issues like airway management, where the complexity of an emergency setting can synergize with patient’s comorbidities and unstable vital signs.^3^

Artificial intelligence (AI) algorithms are automatic and sophisticated methods that recognize complex patterns by combining imaging data with clinical assessments (Table 1). Deep-learning (DL) models aim to represent complex data using more superficial hierarchized structures defined from specific features.^4^ AI has been developed and implemented in health care with the potential to reduce health inequities and improve global health and anesthesiology outcomes.^5,6^ AI tools use big data from electronic health records and complex datasets, integrating different types of information. They identify clusters and extract value to transform large volumes of data into predictive models, thereby enhancing the quality of decision-making quality.^4^ A variety of AI models is machine learning or machine-learning models that use a mathematical formula to predict future events. Although physical airway examination remains an essential tool to identify difficult airways, a multidimensional analysis using machine-learning models to predict difficult airways could be helpful (Table 2). AI and machine-learning represent new advances in airway management.^4^ If machine-learning can predict difficult airways in adults, AI may offer providers real-time clinical decision support during intubation.^4^ Several X-ray machine-learning and DL models have recently been developed and validated to predict difficult airways and identify tracheal tube malpositioning. Other imaging techniques, such as ultrasound (US), computed tomography (CT) scan, and magnetic resonance imaging (MRI), can be helpful for adequately reconstructing airway anatomy.^1^ Nevertheless, the pivotal value of imaging techniques for clinical application may be limited by factors such as radiation, medical costs, equipment requirements, and burden of medical staff can limit their use in clinical practice.^7^

**Table 1.** Example of Tools Used by Artificial Intelligence and Their Applications in Airway Management

Tool	Abbreviation	Definition	Airway outcome
Machine learning	ML	A field of computer science that deals with teaching computers to perform tasks by giving them the ability to study patterns in data, without being explicitly programmed	• Used for airways segmentation with predefined image features • Through the measurement of neck circumference and thyromental distance, it develops a prediction model of difficult laryngoscopy
Deep learning	DL	A type of ML that learns on its own how best to represent data as a hierarchy of concepts, with each concept defined through its relation to simpler concepts.	• Powerful tool used to analyze medical images and to provide human visuals more accurately than laboratory predictions • Model using frontal images of faces and classifying difficult intubations
Artificial neural networks	ANN	network composed of nodes or “neurons” that each perform a computational operation and through which information flows by means of weighted interconnections; to learn to perform a specific task, these weights can be tuned	• With CAD are used to assess the correct positioning of ETTs • Automatically they extract relevant images for airways segmentation • CNN detect laryngeal adductor reflex events in laryngeal endoscopy videos
Big data	BD	A large amount of electronic health data, difficult to manage by traditional software	• The large amount of frontal images of faces used to create AI algorithm
Wearable device(s)	WDs	intelligent devices that can be wearable and not only assist people in pursuing a healthier lifestyle but also provide a constant stream of health care data for disease diagnosis and treatment by actively recording physiological parameters and tracking metabolic status.	• Modern tool designed to detect changes in resistance and pressure during intubation, providing real-time feedback to the operator

Abbreviations: CAD, computer-aided detection; CNN, convolutional neural network; ETTs, endotracheal tubes.

**Table 2.** Principal Scores Used to Evaluate Airways

Score	Name	Definition
LEMON	Look externally	Trauma or face anatomy
	Evaluate 3–3–2 rule	Three fingers between the incisors The mandible length 3 fingers from the mentum to the hyoid bone Two fingers between the hyoid and thyroid
	Mallampati score	>3
	Obstruction or obesity	
	Neck mobility	
Mallampati	1	Visualization of soft palate
	2	Visualization of the uvula
	3	Visualization of only the base of the uvula
	4	Not visualization of soft palate
El-Ganzouri	Mouth opening	><4 cm
	Thyromental distance	<6 cm–>6.5 cm
	Mallampati class	I–IV
	Neck movement	<80°–>90°
	Body weight	<90 kg–>110 kg
	Previous difficult intubation	

**Figure 1.**
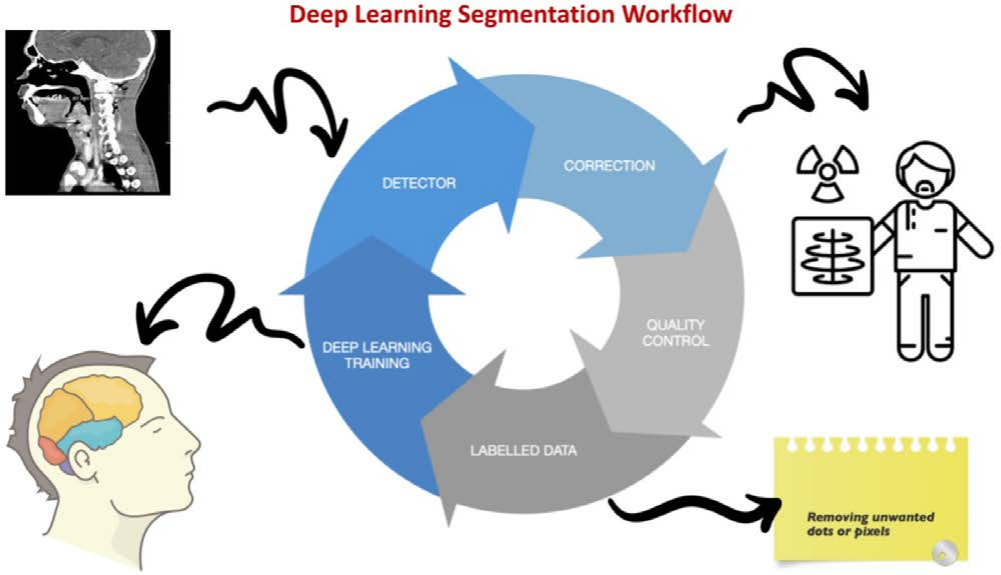
A deep-learning segmentation workflow for difficult airway assessment.

This narrative review aimed to explore the influence of AI models using images and clinical assessment on difficult airway management in clinical practice. Finally, we discuss predictive models for difficult laryngoscopy using machine-learning models and the future application of intelligent intubation devices.

## METHODS

### Search Strategy

Our search strategy was designed to address the following questions:

1. What are the main indications for Tracheal Intubation?
2. Does DL using images and/or clinical assessment improve the diagnostic abilities of anesthesiologists?
3. What are the other applications of AI in this field?
4. Are there any limitations for AI in this field?

To define the aforementioned research questions, we searched in the PubMed database for all published observational studies, randomized trials, systematic reviews, and metanalysis, from inception to January 24, 2024. The following MeSH terms and Boolean operator were used: ((airways management) OR (difficult tracheal intubation)) AND ((artificial intelligence) OR (deep learning) OR (stratification)). We combined the terms and cross-referenced the publications to exclude cases published more than once. We set inclusion criteria to refine the selection of articles based on our subjective assessment of their relevance, novelty, and English language. According to the narrative nature of this review, we did not follow a rigorous methodology for the selection strategy, and we therefore recorded only the total number of publications analyzed (n = 945), the total number of multiple publication cases (n = 72), and the number of publications written in languages other than English (n = 26). A total of n = 847 articles were finally reviewed for titles and abstracts by 2 authors (S.D.R. and D.B.), and n = 31 of them were included for full review. We analyzed the references of each selected article to ensure that other publications missed during the initial search were included.

### Interpretation and Definitions

The relationship between area under the receiver operating characteristic curve (AUROC), positive predictive value (PPV), and negative predictive value (NPV) is complex.^8^ AUROC is used to assess a binary classification model’s efficacy, by differentiating between positive and negative cases.^2,9–12^ An optimal classification is represented by a value of 1, while a random classification is represented by a value of 0.5 for AUROC: higher AUROC values are indicative of improved discriminating between difficult and nondifficult airways, but they do not directly reflect the model’s accuracy in each of its individual predictions. Furthermore, the proportion of difficult airways in the population under test affects the PPV and NPV. PPV and NPV are indicators of how well the prediction model distinguishes between real positive and true negative outcomes.^8^ PPV is the probability that a patient identified by the model as having a challenging airway actually does have a challenging airway. The possibility that a patient who is identified by a model as not having a difficult airway is truly free of a difficult airway is indicated by NPV.^2,8–12^

### Workflow for the DL Segmentation

DL, a subset of machine-learning and representation learning, aims to unravel complex data representations using hierarchized structures defined from specific features.^13^ In health care, DL segmentation provides instrumental analysis of medical images, such as CT scans, MRI scans, and X-rays. DL segmentation involves dividing an image into multiple regions or segments, each representing a distinct part of the image. The workflow for DL segmentation requires careful data preparation, model selection, design, training and validation, testing, and postprocessing to achieve accurate and robust segmentation results:

1. Data collection: New datasets should include both input images and corresponding ground truth (real or true information, provided by direct observation) segmentation maps^14^;
2. Image preprocessing: Implement fixed sequences of operations performed at each image pixel, including resizing the images, normalizing the pixel values, and augmenting the dataset through data augmentation techniques.^14^
3. Model selection and design: Select a suitable DL model for segmentation, such as a convolutional network for biomedical image (U-Net) or mask region-based convolutional neural network (Mask R-CNN) and customize it for your specific use case. This may involve adjusting the architecture, hyperparameters, and loss function.^15^
4. Training: Train the model using the preprocessed dataset by feeding input images into the model and comparing output segmentation maps with the ground truth maps. The goal is to minimize the difference between the predicted and ground truth segmentation maps.^16^
5. Validation: Validate the trained model on a separate validation dataset to assess its generalization ability and detect potential overfitting.^17^ The validation data set provides an unbiased evaluation of the model fit on the training data set.
6. Testing: Test the model on a separate test dataset to evaluate its performance on new, unseen data.
7. Postprocessing: Postprocess the segmentation maps to refine the final results, incorporating techniques such as thresholding, morphological operations, and smoothing.^18^
8. Deployment: Deploy the trained model in a production environment to segment new incoming data.^19^

Figure 1 shows the potential clinical use of DL segmentation for difficult airway assessment.

### AI in X-ray Identification of Difficult Airways

Several X-ray machine-learning and DL models have been based on X-ray imaging and have recently been developed and validated to predict difficult airways.^9,20^ X-rays are commonly used to verify the placement of any lifesaving equipment such as endotracheal tubes (ETTs) and tracheostomy tubes (TTs), to determine the appropriate size of ETT/TT, and to identify complications arising from complex airway management.

X-rays serve as a valuable tool for predicting difficult airways by assessing airway structures, allowing visualization of both bony and soft tissues. This enables the identification of various conditions such as tumors, masses, trauma, abscesses, epiglottitis, tracheal compression, degenerative diseases, or stenosis.^21^ AI models for difficult airway assessment incorporate objective parameters, including hyomandibular distance (≥20 cm) and extension angle (≥38°). Measurements are taken from the anteroinferior point of the upper central incisor tooth to the anteroinferior point of the body of the sixth cervical vertebra, extending anteriorly to the body of the first cervical vertebra.^22^

X-ray is also commonly used to detect malpositioning of ETTs/TTs. Malposition of ETTs occurs in 3% to 14% of cases.^23^ A chest X-ray is typically obtained in the obtained in anteroposterior view, which can limit accuracy in identifying possible malpositioning. Over the last decades, computer-aided detection (CAD) and convolutional neural networks (CNNs) have advanced to assess the correct positioning of ETTs. Lakhani et al^24^ used a deep CNN to demonstrate that X-ray can detect the positioning of ETT with an area under the curve (AUC) of 0.99 for the best-performing network and 0.81 for the most difficult dataset. They classified chest X-rays into 12 categories according to the distance between the ETT tip and carina from 0 to 10 cm and >10 cm.^25^ Harris et al^26^ used a box-based machine-learning model to localize the ETT and carina, measuring the distance in centimeters. This strategy offered information about malpositioning and distance measurement, allowing quantitative information about how many centimeters the tube should be moved to achieve the correct positioning. Chen et al^27^ developed an automatic tool for the detection of ETT and carina using portable chest X-ray using an AI model (Mask R-CNN). DL models demonstrated high interobserver agreement compared to radiologists, typically predicting ETT-carina distance within 1 cm. Notably, the definition of malpositioning may vary among radiologists, influencing data interpretation. AI models exhibit sensitivity and specificity in identifying low ETT locations.^25^ However, pixel segmentation-based models for tube and carina identification did not produce accurate findings (as the CarinaNet model). This suggests the need for implementing a novel segmentation-based model for more precise localization of the tube tip.^28^

### AI in CT Identification of Difficult Airways

CT scans are often used for 3-dimensional reconstruction of airway anatomy. The cross-sectional area from the hard palate to the hyoid bone represents the airway volume. In contrast, the airway length is defined as the vertical distance from the hard palate to the hyoid bone in the median sagittal plane. Airway length was an essential variable in predicting apnea and hypopnea indices in patients with severe obstructive sleep apnea disorder (OSAS; AHI, r = 0.523, *P* < .000), while airway volume was irrelevant. Age (odds ratio, OR =1.084, *P* = .002) and tongue area (OR, 1.002, *P* = .014) were independent risk factors for difficult laryngoscopy.^29^ Han et al^30^ made a 3-dimensional model for the reconstruction of difficult airways. This model helped doctors adopt an individualized management algorithm for difficult airway assessment tailored to a patient’s airway characteristics.

Grimes et al^31^ found that CT scan had a predictive value and PPV of 90.7% and 71.4% to predict difficulties in nasal intubation. The authors found that a nasal diameter of ≤6.3 mm can indicate nasal intubation difficulty. CT scan can also be useful for detecting complications of endotracheal intubation, such as selective intubation of the right bronchus.^32^

CT scans also identify structural abnormalities of the airway tree, such as bronchiectasis (widening of the airways) and thickness of the airway wall, which can be evaluated with thoracic CT segmentation of the airway tree. The airway diameter, wall thickness, and tapering are helpful parameters to quantitatively assess airways on CT scan. The bronchial tree is an intricate, 3-dimensional structure with numerous branches of various sizes and orientations. If airways are manually or semiautomatically segmented, it takes more than 15 hours and 2.5 hours for each scan for manual or semiautomatic segmentation.^32,33^

A complete automated airway segmentation approach can offer precise, simple, and operator-bias-free airway tree segmentation.^33^ Machine-learning algorithms are used for airway segmentation with predefined image features (multiscale Gaussian, Hessian-based features, or image texture features with local binary patterns), allowing more complete airway tree predictions with fewer false positives. However, the limitations of these methods include high dependency on the image features used to train the classifier and the long time needed. Current and more recent methods include CNNs, which automatically extract relevant images for airway segmentation.^33^

### AI in MRI Identification of Difficult Airways

AI can be used to assist in the identification and assessment of difficult tracheal intubations using MRI.^10^ Despite the ability to safely image soft tissue, MRI inherently suffers from low signal-to-noise ratio (SNR), which causes long scan times,^34^ but it is also expensive and time-consuming. However, MRI is a good tool for evaluating the influence of airway soft tissue structure on difficult laryngoscopy. One potential application of AI in this context is the use of image recognition algorithms to analyze MRI scans of the neck and airway to identify anatomical features that may make intubation challenging. For example, AI algorithms can be trained to identify the presence of anatomical variants such as a high-arched palate, a narrow oropharynx, or a short neck, which can increase the difficulty of intubation. Additionally, AI can be used to analyze patient data, such as their medical history, age, weight, and previous intubation experiences, to help predict the likelihood of difficult intubation.

Münster et al^35^ reported in 142 patients a correlation between difficult laryngoscopy and the anatomical position of the vocal cords concerning the cervical vertebrae as assessed by MRI. Unfortunately, beyond this study, there is very little research evidence to support the effectiveness of MRI in assessing difficult airways.^36^

### AI in Facial Image Identification of Difficult Airways

Cuendet et al^11^ described a model for the identification of difficult endotracheal intubation by analyzing the faces of 900 patients in various positions (open mouth, protruding tongue, upturned gaze, and laterally rotated head) to identify typical morphological traits and create a fully automated method for difficult airway prediction with an AUC 0.81.

Tavolara et al^2^ applied AI to this analysis system and developed a DL model using frontal images of faces; they created a large database with images of celebrities to train 11 CNNs on 11 facial regions to obtain the predictive model. The model showed an AUC of 0.7105, with high sensitivity and low specificity. This study represents only a preliminary stage in developing a method for clinical application of AI at the bedside. Nevertheless, given the considerable amount of data on which it is based, it could provide an excellent basis for future models.

Hayasaka et al^10^ applied DL and CNNs to create an AI model by classifying difficult intubations from images of patients’ faces. Through an observational study of 205 patients, they used photographs of each patient in 16 different positions. They collected age, gender, body mass index (BMI), comorbidity, upper lip bite test, Mallampati score, jaw length, dentition, interincisive distance, neck movement, and thyromental distance. Patients who presented with a Cormack 3 or 4 had a 26.7% rate of difficult intubations. Applying 2 DL methods and a dataset as a test, they identified the best predictive model with an AUC of 0.864, accuracy of 80.5%, sensitivity of 81.8%, and specificity of 83.8%. This model analyzes images of the patient’s face placed in a supine position with the airway in the neutral position, and the mouth closed, allowing to analyze the shape of the neck and to identify difficult and nondifficult intubations.

These predictive models of facial analysis can be easily used during preoperative assessment and can enrich the anesthetist’s capabilities, providing fast and safe results. However, these algorithms require numerous images of the patient’s face, in various positions. The use of other new technologies combined with AI, as new mobile devices, could optimize the usability of these techniques. An example of this technological combination is the study of Mendoza et al,^37^ in which the authors developed a machine-learning system based on 2 types of photographs taken from patients, in frontal position with the mouth open, and in lateral position with the head in vertical extension, via a smartphone with a camera. This could lay the foundations for rationalizing the collection of preoperative images by creating registries that can be accessible at each perioperative stage and in critical care setting.

### Predictive Model Using Clinical Assessment For Difficult Airways Using Machine-Learning

Zhou et al^9^ used 10 DL and machine-learning algorithms in patients undergoing thyroid surgery and identified as the 5 most important factors in determining a difficult airway: age, gender, weight, height, and BMI. In a retrospective analysis of 500 patients, 48 of them showed a difficult laryngoscopy, and the best predictive model was found to be Gradient Boosting with an AUC >0.8, an accuracy >90%, and a precision of 100%.

The same group had previously dealt with obese patients using at least 6 machine algorithms and identified a model termed Xgbc as the best model with an accuracy >80%.^38^

On the contrary, Jong Ho Kim et al^38^ developed a prediction model for difficult laryngoscopy using machine-learning through neck circumference and thyromental distance measurements. Individually, these were not good predictors, but put together in the BRF machine-learning algorithm they achieved an AUROC of 0.79 with a sensitivity of 90%.^39^ These data can be further improved by entering new data, variables, and model combinations.

Langeron et al^40^ demonstrated that computer-based augmentation models are better than other conventional methods and identified the after parameters as the best predictors of difficult intubation: BMI, age, Mallampati grade, thyromental distance, mouth opening, macroglossia, gender, receding chin, and snoring. Moustafa et al^12^ instead chose 9 predictors of difficult intubation (interincisive distance, thyromental distance, modified Mallampati score, upper lip bite test, joint extension) and created a predictive DL method with an AUROC of 0.79.

Yamanaka et al^20^ used many parameters to be entered into 7 machine-learning algorithms: LEMON parameters (age, gender, estimated height and weight, and BMI), preintubation vital parameters (blood pressure, heart rate, respiratory rate, saturation), and Glasgow coma score. First-attempt intubation was also analyzed, which was mainly influenced by the use of laryngeal pressure, and the models developed with machine-learning performed decisively better (*P* > .01, sensitivity of 0.67, specificity of 0.70, PPV of 0.09, and NPV of 0.98). Although these predictive models demonstrate excellent performance in research, enhanced by the addition of data, implantation into clinical practice has still not happened.^41^

### Intelligent Intubation Devices

An intelligent initiation device (IID) is a modern tool designed to detect changes in resistance and pressure during intubation, providing real-time feedback to the operator. Some examples of intelligent intubation devices include video laryngoscopes, automated intubation devices, and wireless endoscopy systems.^42^

Visualization techniques and AI can improve the accuracy rate of tracheal intubation procedures by providing users with real-time guidance and feedback and automatically adjusting the intubation device based on the patient’s anatomical features. These devices are equipped with AI algorithms to improve accuracy and success rate. For example, an intelligent ETT may be designed to adjust its shape or size based on the patient’s anatomical features or to detect and correct any tube misplacement during intubation. They can help reduce the risk of complications such as esophageal intubation or damage to the vocal cords.

**Figure 2.**
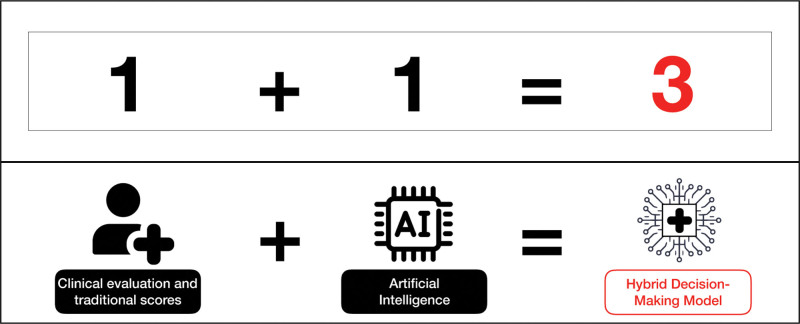
Clinical evaluation and traditional scores associated with AI led to hybrid decision-making which is more than the simple sum of these 2 components. AI indicates artificial intelligence.

AI algorithms can automatically identify the pharynx and trachea based on a video laryngoscopy. Deep CNNs can automatically detect laryngeal adductor reflex events in laryngeal endoscopy videos.^43^ A user-friendly software tool for automated quantification of vocal fold movements from previously recorded video-laryngoscopy examinations has been created, termed automated glottic action tracking by artificial intelligence (AGATI). This tool may be helpful in the diagnosis and tracking of outcomes of vocal fold movement disorders.^44^ AI algorithms can also assist doctors during intubation and positioning, allowing automatic identification of blood vessels and the trachea based on fiberoptic bronchoscopy.^36^ Intelligent intubation devices are becoming increasingly common in hospitals and other medical settings due to their ability to improve the accuracy and safety of intubation procedures. However, they should always be used under the supervision of a trained health care professional to ensure proper placement of the ETT and minimize the risk of complications. Figure 2 shows how AI associated with traditional evaluation of difficult airways can lead to a new model of decision-making, which is more than the sum of the 2 components.

### Future Perspectives

Airway management for difficult intubation is a complex and multifactorial process. Machine-learning models using either images or clinical assessments demonstrated significantly higher sensitivity (approximately 80%–90%) and specificity (approximately 90%–100%)^2,9–12^ than conventional airway examination methods for unanticipated difficult intubation (low sensitivity of 20%–62% and specificity of 82%–97%).^2^ Despite the unquestionable growing utility and efficacy of machine-learning models for supporting the clinical practice of anesthesiologists^45^ and critical care physicians in difficult airway assessment, models of AI have some limitations that need to be addressed. First, these models have mostly been developed in specific and predefined scenarios that might not reflect situations of unpredictable difficult intubation or emergency laryngoscopy. Indeed, some studies included only patients who were scheduled for surgery. As a result, compared to an emergency scenario, the elective setting does not reflect all possible cases of unpredictable difficult intubation or intensive care unit (ICU) setting, where many other factors can influence airway management.^10^ Second, self-reporting and measurement bias may affect the data, for instance, underreporting difficult airways. Since procedural expertise is difficult to define and assess in real-world circumstances, they may have lacked specific information on each beholder’s expertise and preference.^20^ Third, in some studies, only patients of specific ethnic categories were included (ie, Asian, European), limiting the generatability on a larger scale.^46^

**Figure 3.**
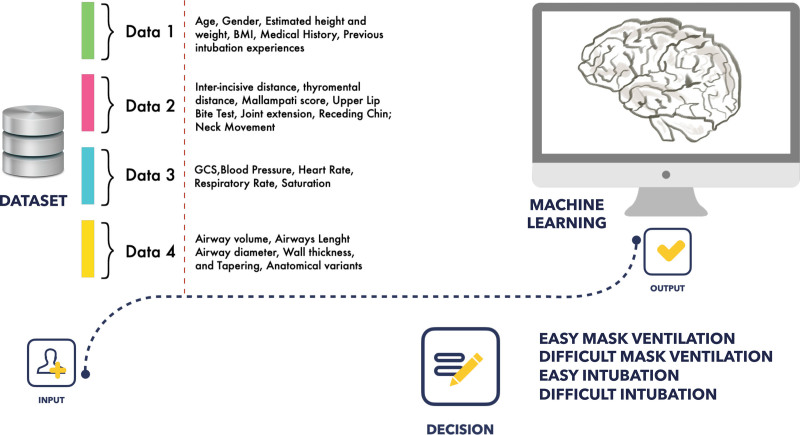
Possible approach to airway management using AI and machine-learning algorithms. AI indicates artificial intelligence

The main challenge of finding an effective AI model for difficult intubation is to capture multiple confounders that cannot be easily extrapolated by a simple machine but should be contextualized with the beholder’s needs, experience, and expectations. This may include the effective laryngeal view, operator experience, patient’s vital signs, physiological status, appropriate preoxygenation, choice of initial technique, and many more. These important factors for difficult airway management are still poorly considered and difficult to capture in current AI models, suggesting the need for implementation. An ideal machine-learning model would capture all known correlates of difficult intubation from patient instability to radiographic exams to physical characteristics like neck circumference to environment. AI models should be designed to support human decision-making without replacing physicians’ decision, for example, for converting a planned asleep intubation into awake intubation, or to facilitate the choice of using video laryngoscopy rather than direct laryngoscopy for the first intubation attempt. Table 1 includes the current available AI models for difficult intubation and the study outcomes. Further studies are needed to design new AI models for difficult airways in specific settings (ie, emergency, ICU), populations (ie, Afro-American, European, mixed, etc.), and accounting for confounding variables (ie, hemodynamic stability, operator experience, etc.).^3^ Figure 3 shows a possible approach to airway management using AI and machine-learning algorithms.

## CONCLUSIONS

Techniques using AI and DL models offer promise in difficult airway management. What is next needed are large, multicenter trials focused on the best available technology as a prelude to including these in clinical pathways. Specifically, those patients undergoing preoperative head and neck imaging could have their degree of difficulty of intubation predicted, based on the data already available, and assessing this against actual outcomes would be insightful. A combined approach with images and clinical assessment is needed.

## ACKNOWLEDGMENTS

We thank Professor Paolo Pelosi, Anesthesia and Intensive Care, San Martino Policlinico Hospital, IRCCS for Oncology and Neuroscience, Genoa, Italy for creating this strong network, and for getting us together.

## DISCLOSURES

**Name:** Silvia De Rosa, MD.

**Contribution:** This author helped in conception and design of the article, interpretation of data, drafting and editing of the article, and read and approved the submitted version.

**Name:** Elena Bignami, MD.

**Contribution:** This author helped in conception and design of the article, interpretation of data, drafting and editing of the article, and read and approved the submitted version.

**Name:** Valentina Bellini, MD.

**Contribution:** This author helped in conception and design of the article, interpretation of data, drafting and editing of the article, and read and approved the submitted version.

**Name:** Denise Battaglini, MD, PhD.

**Contribution:** This author helped in conception and design of the article, interpretation of data, drafting and editing of the article, and read and approved the submitted version.

**This manuscript was handled by:** Narasimhan Jagannathan, MD, MBA.
